# Influence of the Tumor Microenvironment on NK Cell Function in Solid Tumors

**DOI:** 10.3389/fimmu.2019.03038

**Published:** 2020-01-21

**Authors:** Ombretta Melaiu, Valeria Lucarini, Loredana Cifaldi, Doriana Fruci

**Affiliations:** ^1^Paediatric Haematology/Oncology Department, Ospedale Pediatrico Bambino Gesù, Rome, Italy; ^2^Department of Biology, University of Pisa, Pisa, Italy; ^3^Academic Department of Pediatrics (DPUO), Ospedale Pediatrico Bambino Gesù, Rome, Italy

**Keywords:** natural killer cells, tumor microenvironment, solid tumors, immune checkpoint inhibitors, cellular metabolism, cancer stem cells, hypoxia, adoptive transfer of NK and CAR-NK cells

## Abstract

Natural killer (NK) cells are a population of innate lymphoid cells playing a pivotal role in host immune responses against infection and tumor growth. These cells have a powerful cytotoxic activity orchestrated by an intricate network of inhibitory and activating signals. The importance of NK cells in controlling tumor growth and in mediating a robust anti-metastatic effect has been demonstrated in different experimental mouse cancer models. Consistently, high density of tumor-infiltrating NK cells has been linked with a good prognosis in multiple human solid tumors. However, there are also tumors that appear to be refractory to NK cell-mediated killing for the presence of an immunosuppressive microenvironment affecting NK cell function. Immunotherapeutic strategies aimed at restoring and increasing the cytotoxic activity of NK cells in solid tumors, including the adoptive transfer of NK and CAR-NK cells, are currently employed in preclinical and clinical studies. In this review, we outline recent advances supporting the direct role of NK cells in controlling expansion of solid tumors and their prognostic value in human cancers. We summarize the mechanisms adopted by cancer cells and the tumor microenvironment to affect NK cell function, and finally we evaluate current strategies to augment the antitumor function of NK cells for the treatment of solid tumors.

## Introduction

Natural killer (NK) cells are a specialized population of innate lymphoid cells (ILCs) that mediates cytotoxic functions against damaged, infected, and pre-malignant cells through an intricate network of signals that allow for rapid activation (1). NK cell cytotoxicity is mainly regulated by the secretion of effector molecules (IFN-γ, TNF-α, NO, IL-2, IL-12, IL-15, IL-18, and IL-21), and the interplay between inhibitory and activating signals originating at the cell surface from NK cell-inhibitory receptors (NK-IRs) and NK cell-activating receptors (NK-ARs), respectively. NK-IRs promote the effector function upon interaction with ligands expressed on normal and healthy cells. Conversely, NK-ARs recognize ligands encoded by pathogens (2), or induced by cellular stress during viral infections (3), or cellular growth factors (4). The downregulation of inhibitory ligands and the expression of ligands for NK-ARs on cancer cells can trigger NK cells to kill them and secrete cytokines, such as IFN-γ and TNF-α. Thus, blocking interaction between NK-IRs and their ligands or enhancing NK-ARs–ligand binding may represent a promising strategy to generate an antitumor activity.

NK cells in the peripheral blood, spleen, and bone marrow can infiltrate other tissues. In human, two distinct subsets of NK cells have been identified based on CD56 expression levels. The CD56^bright^ NK cell subset, representing the majority of NK cells in the peripheral blood, is specialized in the secretion of cytokines and chemokines in response to IL-12, IL-15, and IL-18, and in the regulation of adaptive immunity. The CD56^dim^ NK cell subset is both cytotoxic and cytokine-producing and expresses high levels of CD16 (also known as FcγRIII), which is responsible for antibody-dependent cellular cytotoxicity (ADCC). The recruitment of NK cells in the inflamed tissues is regulated by the expression of several chemokine receptors, including CXCR3 that binds to the tumor-derived chemokine ligands CXCL9, CXCL10, and CXCL11 (5). Although the presence of tumor-infiltrating NK cells confers a favorable outcome in many tumors, in others, their function is impaired by soluble modulators secreted in the tumor microenvironment (TME). In this Review, we discuss recent evidence for a direct role of NK cells in controlling tumor expansions and summarize the mechanisms adopted by tumor cells and the TME to affect NK cell functions in solid tumors. Finally, we evaluate novel therapeutic strategies to enhance the antitumor function of NK cells for the treatment of solid tumors.

## Role Of Nk Cells In The Immunosurveillance Of Solid Tumors

NK cells control tumor growth by interacting directly with tumor cells or affecting the function of other populations of innate and adaptive immunity in the TME. The importance of NK cells in antitumor immunity has been established in different experimental mouse tumor models.

Depletion of NK cell populations prior to tumor transplantation has been shown to cause a more aggressive phenotype with metastatic tumors (6–8). Specifically, the use of mutant mice with developmental and functional alterations of NK cells allowed a better understanding of the role of these cells in antitumor immunity. NCR1/NKp46 is directly involved in the killing of melanoma and Lewis lung carcinoma cells and in the formation of metastases (9). Indeed, NCR1^−/−^ mice underwent a more aggressive tumor development compared to wild type (WT) mice. Mice knockout for Mcl1, which is required to sustain the *in vivo* survival of NK cells, were characterized by the total absence of NK cells and a rapid development of metastatic melanomas (10). A similar observation was reported in IL-2rg^−/−^ and TLR3^−/−^ mice (11, 12). TLR3 is known to limit B16F10 lung metastasis through the production of IFN-γ by NK cells. The lack of TLR3 signaling downregulates NK cell function following cytokine stimulation, leading to defective immune responses unable to constrain metastatic diseases (12). DNAM-1^−/−^ mice developed fibrosarcoma and papilloma in response to chemical carcinogens significantly more frequently than WT mice (13). Tbx21, also known as T-bet, is a transcription factor involved in the differentiation of NK cells. Tbx21^−/−^ mice injected intravenously with melanoma or colorectal carcinoma cells were more susceptible to metastasis formation compared to WT mice (14). The ability of NK cells to invade the primary tumors and migrate in the metastatic site is dependent on the heparanase. Mice lacking heparanase specifically in NK cells (Hpse^fl/fl^ NKp46^−iCre^ mice) were more susceptible to develop lymphoma, metastatic melanoma, prostate carcinoma, or mammary carcinoma when challenged with the carcinogen methylcholanthrene (15). These observations suggest that NK cells play a prominent role in controlling tumor growth and in mediating a robust anti-metastatic effect.

Further evidence for the role of NK cells in controlling tumor development and dissemination derived from the ability of these cells to target and eliminate cancer stem cells (CSCs), a subset of cells with self-renewal ability involved in the generation and evolution of tumors (16). CSCs exhibit a typical surface expression profile consisting of low levels of MHC class I, CD54 and PD-L1, and high expression of CD44 (17). The susceptibility of CSCs to NK cell-mediated killing has been reported in different tumor models (18, 19). An *in vivo* study reveals that activated NK cells transferred in NSG mice harboring orthotopic pancreatic cancer xenografts were able to preferentially kill CSCs, leading to a significant reduction of both intratumoral CSCs and tumor burden (20). Additionally, in colorectal cancer, CSCs upregulated the NK-ARs NKp30 and NKp44 and were susceptible to NK cell-mediated killing (19). Similarly, glioblastoma-derived CSCs showed an increased susceptibility to NK cell killing by both allogeneic and autologous IL-2 and IL-15 activated NK cells (21). Melanoma cell lines with CSC features exposed to IL-2-activated allogeneic NK cells showed an increased susceptibility to NK cell-mediated killing through upregulation of the DNAM-1 ligands, such as PVR and Nectin-2 (22). Breast cancer CSCs showed sensibility to IL-2- and IL-15-treated NK cells and increased expression of NKG2D ligands, such as ULBP1, ULBP2, and MICA (23).

CSCs are also considered an important source of resistance to standard anti-cancer therapies. Following chemotherapy and radiation therapy treatments, CSCs upregulate ligands for NKG2D such as MICA and MICB, resulting in an increase of NK cell cytotoxicity (24, 25).

NK cells are able to target and shape CSC-undifferentiated tumors, thereby leading to a selection of a differentiated tumor subset (26). After selection, NK cells down-modulate their surface receptors, lose their cytotoxicity, and become anergized, but continue to produce IFN-γ and TNF-α, which drive differentiation of the remaining stem cells. This results in an increased expression of MHC class I, CD54, and PD-L1 and reduction of CD44 on CSC surface. These cells exhibit a decreased proliferation rate, inability to invade or metastatize and increased susceptibility to chemotherapeutic and radio-therapeutic agents (26, 27).

Despite the role of NK cells in targeting CSC/undifferentiated tumors, some authors have highlighted an association between the stage of differentiation and sensitivity to NK cell-mediated cytotoxicity. Studies conducted on patients with pancreatic tumors or oral squamous carcinoma stem cells revealed that although CSCs/undifferentiated tumors were susceptible to NK cell-mediated cytotoxicity, they remained significantly resistant to chemotherapeutic and radiotherapeutic agents. Conversely, differentiated tumors grew slower and were resistant to primary NK cell-mediated cytotoxicity with high susceptibility to chemotherapeutic and radiotherapeutic agents (27, 28).

The role of NK cells in controlling tumor growth is also supported by several evidence in human specimens. The first studies date back to the late 1980s (29–31). Several authors have studied the phenotype of circulating NK cells and their cytotoxic activity in cancer patients compared to healthy donors (32). Other authors have investigated the prognostic role of tumor-infiltrating NK cells (33), but due to the lack of immunohistochemical markers that unambiguously identify NK cells, current information is limited. Different methods are used to attribute a prognostic value to specific immune cell subsets. The most reliable is the one that evaluates survival analysis stratifying the subjects according to the median cutoff value for density of immune cell subsets.

Denkert et al. showed that a higher density of NK cells is an indicator of good prognosis in breast cancer (33). A pooled analysis of 3,771 patients treated with neoadjuvant therapy revealed the presence of intratumoral immune cell types, including NK cells, significantly associated with better prognosis of HER2-positive and triple negative breast cancer patients (33). Trastuzumab treatment was associated with a significant increase of tumor-infiltrating NK cells and expression of granzyme B and TiA1 in breast cancer compared with controls (34). Moreover, the expression of NCR1 and other NK cell-associated genes, i.e., CD1d, DNAM-1, CRTAM, CD96, and NCR3/NKp30, was associated with prolonged disease-free survival (DFS) of breast cancer patients (34). High number of intratumoral NK cells was considered an optimal biomarker of response to the anti-HER2 antibody-based treatment. Indeed, the presence of these cells was significantly associated with a complete response and extended DFS (35). The same authors showed that the number of circulating CD57^+^ NK cells was inversely correlated to tumor-infiltrating NK cells and predicted resistance to HER2-specific antibody treatment in HER2-positive primary breast cancers (36). From a clinical perspective, baseline screening for high circulating CD57^+^ NK cells could be implemented for the identification of patients with primary resistance to neoadjuvant treatment with HER2 therapeutic antibodies, complementing the positive predictive value of tumor-infiltrating NK cells in diagnostic biopsies (27).

Melanoma patients were characterized by expansion of CD56^dim^CD57^dim^CD69^+^CCR7^+^KIR^+^ tumor-infiltrated NK cells (37). These cells showed robust cytotoxicity against autologous tumor cells compared to those derived from tumor-free ipsilateral lymph nodes of the same patients (37). Melanoma cells isolated from metastatic lymph nodes were efficiently lysed by circulating NK cells expressing high levels of NKG2D, NKp30, DNAM-1, and CD62L (38).

Recently, the prognostic role of NK cells has been investigated through the expression of genes specific for NK cell function. NK cell gene signatures were associated with a strong survival advantage for metastatic melanomas (39). More recently, Barry et al. defined a clear link between NK cells and dendritic cells (DC), demonstrating that NK cells produce FLT3LG cytokine that controls the intratumoral level of BDCA-3^+^ DCs. The authors also showed that the high expression of NK and DC gene signatures was associated with a better OS in two independent datasets of melanoma patients (40).

High levels of tumor-infiltrating NK cells have been associated with a good prognosis also in other human cancers, including gastrointestinal stromal tumor (41–43), neuroblastoma (44), head and neck cancer (45), and prostate cancer (46). A more complex and heterogeneous landscape has been outlined for other types of neoplasms. A gene signature able to predict tissue NK cell content and prognosis of renal cell carcinoma patients was proposed (47). Two distinct groups of renal cancer patients have been identified based on the level of tumor-infiltrating NK cells (48). In both groups, NK cells were not cytolytic but differed for CD16 expression levels. NK cells from tumors with high NK cell content (>20% of the lymphocyte population) were CD16^bright^, whereas those from tumors with low NK cell content (<20%) were CD56^dim^. *In vitro* experiments showed that unlike the low-NK group, the high-NK group was able to acquire cytotoxic function against K562 cells (48). An early study reported that low NK cell cytotoxicity was predictive of colon cancer recurrence, independently of other prognostic factors (30). These data were confirmed in patients with metastatic colorectal cancer treated with the mAb 17-1A (49). Recently, density and tissue distribution of NK cells were investigated in 112 primary colorectal cancer samples (50). Despite high concentration of chemokines known to promote NK cell infiltration, colorectal cancers were only scarcely infiltrated by these cells as compared to normal mucosa, thus suggesting the presence of soluble factors in the TME preventing NK cell infiltration.

The tissue distribution of NK cells was studied in different subtypes of lung cancer. A high number of intratumoral CD57^+^ NK cells, as evaluated by IHC analysis, was significantly associated with a better clinical outcome in squamous cell lung cancer patients (51). A gene expression study conducted on 148 blood samples discovered valid prognostic innate immune markers (52). Among the screened genes, the enhanced expression of *NCR3* was associated with better overall survival (OS) in non-small cell lung cancer patients. More recently, a low number of circulating NKp46^+^ CD56^dim^ CD16^+^ NK cells was significantly associated with a better OS in small cell lung cancer patients. Intratumoral NK cells were less cytotoxic in non-small cell lung cancer patients, as compared to circulating NK cells or those derived from normal lung tissues (53). Of note, NK cells were located in the tumor stroma not in direct contact with cancer cells. Further evidence indicate that NK cells are very rare within human non-small cell lung cancer and that those infiltrating tumor tissues resemble the circulating CD56^bright^ NK cells (54). More recently, Lavin et al. found that NK cells are the least abundant immune cell population within lung adenocarcinomas and that those expressing CD16 are dramatically reduced in tumors as compared to normal tissues (55). This subset of NK cells is less cytotoxic, expressing low levels of granzyme B, IFN-γ, and CD57.

A reduced number of CD56^dim^CD16^high^ NK cells was detected in the liver tumor area as compared to non-neoplastic area (56). These cells also showed an impaired cytotoxic ability. In general, high density of NK cells within intratumoral region of hepatocellular carcinoma showed better OS and DFS. Conversely, the few NK cells infiltrating advanced hepatocellular carcinoma exhibited attenuated capacities for cytokine productions. These evidence indicate that infiltration of functional NK cells in hepatocellular carcinoma tissues may represent the host reaction to cancer and that TME impairs NK cell function during disease progression (57).

Recently, a new subset of liver-resident NK cells characterized for expression of CD49a has been discovered (58). These cells are abundant in the peritumoral area of hepatocellular carcinoma and are characterized by the expression of PD1, CD96, and TIGIT. The accumulation of these cells in liver tumors was correlated with poor prognosis, thus suggesting a role of this NK cell subset in the hepatocellular carcinoma development (58).

In prostate cancer, a strong immunosuppressive microenvironment impairs NK cell function at multiple levels. Indeed, NK cells that infiltrated the cancer prostate tissues were mainly CD56^+^ and displayed an immature, but activated phenotype with low or no cytotoxic potential. The authors found that TGF-β is highly secreted into the TME and provided an immunosuppressive effect on NK cells. Co-culture experiments revealed that tumor cells induced expression of inhibitory receptors downregulating that of the activating receptors NKp46, NKG2D, and CD16 on NK cells, thus preventing their recognition (46). Similarly, endometrial cancer was poorly infiltrated by NK cells. These cells, when present, expressed co-inhibitory molecules, such as TIGIT and TIM-3, proportionally with the severity of the disease, thus suggesting an important role of the TME in reducing recruitment of functional NK cells to the tumor site (59).

Overall, many evidences indicate that NK cells, although representing an extremely rare subset of immune cells, are able to invade some solid tumors and, when functionally activated, are associated with good prognosis. However, in several cases, the picture is much more complex, with contradictory results, and no or negative effects on patient's prognosis. Different factors contribute to attenuate the antitumoral properties of NK cells, thus providing the need to integrate the study of NK cell density, with equally important elements including phenotype and localization with respect to stroma and parenchyma tumor cells and other immune cell populations. Discordant results obtained in certain tumors could be derived from the methods used to unequivocally identify NK cells, the type of tissue, or the tumor phase (60). The inability of NK cells to limit tumor growth and improve patient survival may result from the presence of an immunosuppressive TME. Indeed, TME renders solid tumors particularly refractory to NK cell killing through a plethora of strategies ranging from preventing the recruitment of intratumoral NK cells, to confining them, when present, to the stromal part of the tissue, or rather attract the recruitment of non-cytotoxic NK cells (CD56^bright^CD16^low/neg^) in the tumor bed.

## Immunosuppressive Properties Of The Tme On Nk Cells

**Figure 1:**
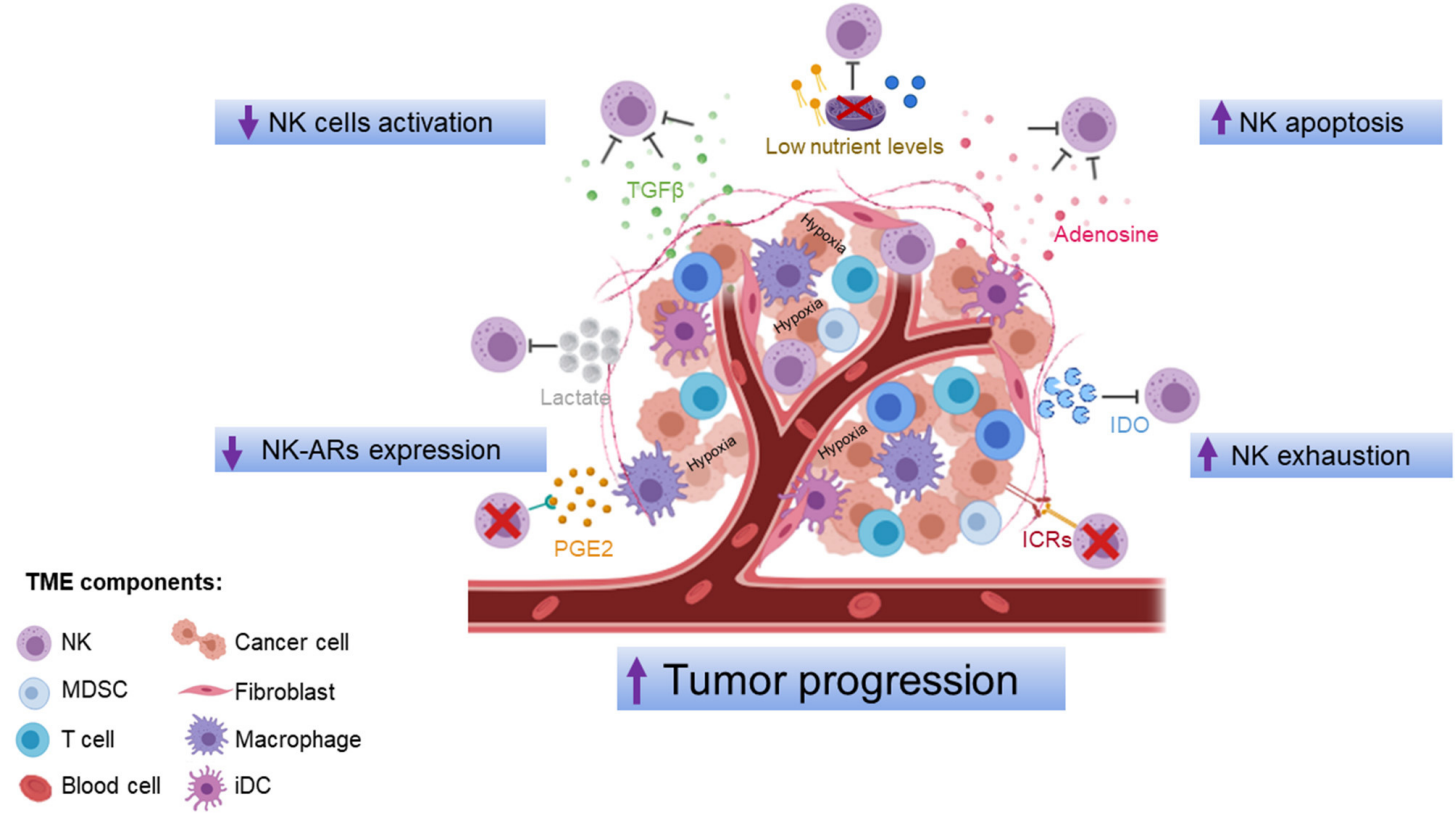
Mechanisms of NK cell dysfunction in the tumor microenvironment. The tumor microenvironment inhibits NK cell function via production of soluble modulators, low nutrient levels, and hypoxic conditions that negatively regulate maturation, proliferation, activation, and effector function of NK cells. ICRs, immune-checkpoint receptors; IDO, indoleamine 2,3-dioxygenase; PGE2, prostaglandin E2; TGF-β, transforming growth factor-β; iDC, intratumoral dendritic cells; MDSC, myeloid-derived suppressor cells; NK, natural killer cells. The figure was performed with https://biorender.com/.

TME, composed of cancer cells, fibroblasts, endothelial cells, and immune cells, provides conditions that promote tumor progression. Accumulating evidences indicate that TME produced soluble modulators that negatively regulate maturation, proliferation, and effector function of NK cells (Figure 1). These immunosuppressive factors may act either directly on NK cells or indirectly by stimulating other immune cells, such as antigen-presenting cells (APC), regulatory T cells (Tregs), and myeloid-derived suppressor cells (MDSC), to produce additional immunosuppressive molecules.

TGF-β is a cytokine produced in the TME by tumor cells, Tregs, MDSCs, and other stromal cells. This cytokine is known to inhibit both the expansion and function of effector cells and to promote the proliferation of Tregs (61). TGF-β impairs NK cell function directly or indirectly by cell–cell contact between NK and other cytokine-producing cells (62, 63). As a direct effect, TGF-β limits NK cell cytotoxicity and IFN-γ production by inhibiting the T-bet transcription factor (SMAD3) (64) and downregulating the expression of NKp30 and NKG2D, and its ligand MICA in cancer patients (65–67). In colorectal cancer and lung cancer, downregulation of NKG2D has been associated with increased serum levels of TGF-β (68). The levels of DNAX activating protein 12 kDa (DAP12), a crucial signaling adaptor of NKG2D in human NK cells, were downregulated by TGF-β-induced miR-183 (69). In an orthotopic liver cancer model, TGF-β was shown to bind on the cell surface of MDSCs and inhibits NK cell function (70). TGF-β is also able to inhibit NK cell function by targeting the serine and threonine kinase mTOR, a crucial signaling integrator of pro- and anti-inflammatory cytokines, such as interleukin-15 (IL-15), in both murine and human NK cells (71). Interestingly, TGF-β is able to transdifferentiate NK cells into ILC type 1 (ILC1), which are missing of cytotoxic functions (72, 73). TGF-β is also able to dampen CD56^dim^ recruitment and favor that of CD56^bright^ (74). It contributes to modulate chemokine repertoire reducing the expression of those attracting CD56^dim^ NK cells (CXCL2, CX3CL1, CXCL1, and CXCL8), and increasing that of chemokines (CXCL9, CXCL10, CXCL19, and CCL5), driving migration of CD56^bright^ NK cells (60, 74). In ovarian (75) and lung (76) cancer patients, CD56^bright^/CD16^low^ represents the predominant NK cell population in the TME. Moreover, the presence of intratumoral tertiary lymphoid structures (TLS) in lung cancer drives the expression of chemokines normally secreted in secondary lymphoid organs (CCL19, CCL21, etc.), thus preferentially attracting non-cytotoxic CD56^bright^ NK cells at the tumor site (76). Based on these findings, TGF-β may be considered a target for enhancing NK cell-mediated antitumor immunity. Currently, inhibition of TGF-β signaling in preclinical studies (77–83), including those based on the combined use of immune checkpoint inhibitors, such as anti-PD-L1 (84–86), as well as in clinical trials (87–89), represents a promising antitumor approach supported mainly by the analysis on CD8^+^ T cells. Interestingly, the use of anti-TGF-β in solid tumors allows the accumulation of NK cells, the increased production of IFN-γ, and the restoration of NKG2D (90).

Another crucial factor leading to dysfunctional NK cells in cancer is the impaired cellular metabolism (91–93). TME is known to be very poor in nutrients, such as glucose and glutamine, very important for NK cells (94–97). While much attention has been focused on T cells and macrophages (98–101), little is known about the NK cell metabolism. In humans, increased glycolysis and oxidate phosphorylation (OXPHOS) stimulate CD56^bright^ NK cells to produce IFN-γ (102). In mice, steady-state NK cells favor OXPHOS, rather than glycolysis, as the primary metabolic pathway. Conversely, activated NK cells induced by IL-2/IL-12 have a greater preference for glycolysis. Glycolysis is an important metabolic process for NK cell function, and in general, it is increased in activated NK cells. A recent study showed that lung tumors upregulate the expression of the gluconeogenesis enzyme fructose bisphosphatase 1 (FBP1) in tumor-infiltrating NK cells through a mechanism involving TGF-β (92). This leads to NK cell dysfunction by inhibiting both glycolysis and the viability of NK cells. The authors showed that pharmacological inhibition of FBP1 can revert the dysfunctional phenotype of NK cells during tumor promotion, but not during tumor progression. Indeed, similarly to tumor-infiltrating T cells (103, 104), NK cells in the mouse lung cancer are functional during tumor initiation, while assume a mild dysfunctional state during tumor promotion that can be reverted by pharmacological FBP1 inhibition. Subsequently, NK cells evolve into an irreversible dysfunctional state that can no longer be rescued by FBP1 inhibition (93). Other molecules that can potentially inhibit NK cell metabolism include two physiological products of enzymatic cholesterol oxidation, 25-hydroxycholesterol and 27-hydroxycholesterol, that inhibit the activation of SREBP transcription factors, key regulators of NK cell metabolism (105–107). Further evidence supporting the role of the impaired cellular metabolism in leading to dysfunctional NK cells come from studies on obesity in human and murine models. NK cells from obese mice and humans are less responsive to tumor target cells in terms of cytotoxicity and production of IFN-γ, granzyme B, and perforin (108). This is because NK cells fail to engage a metabolic response when stimulated with cytokines and have reduced metabolic rates, compared to lean counterparts (108). This metabolic dysfunction has been linked to peroxisome proliferator-activated receptor (PPAR)-driven lipid accumulation in NK cells leading to altered gene expression, downregulation of mTORC1 and MYC signaling, and decreased rates of glycolysis and OXPHOS (108). NK cells from obese mice and humans fail to kill tumor cells for their inability to form synapses with them, an event that is highly energy consuming (108).

The production of prostaglandin E2 (PGE2) in the TME is another mechanism adopted by tumors to selectively suppress the effector function of NK cells (109–112). PGE2 produced by tumor cells, tumor-associated macrophages, and stromal cells (113–115) represents a key regulator of the NK cell activity for mesenchymal stem cell (MSC)-mediated regulation of NK cell activity (116), tumor-derived MSCs (T-MSCs) (117), and MDSCs (118). In addition, PGE2 produced by the thyroid cancer cell microenvironment suppresses NK cell cytotoxicity (119). PGE2 downregulates the expression of NKp30, NKp44, NKp46, and NKG2D by binding to E-prostanoid 2 (EP2) and EP4 receptors on NK cells (112), via a common cAMP-PKA signaling (120), thus resulting in the inhibition of cytotoxicity (116, 121). In PGE2-producing tumors, NK cells were less abundant and impaired in their ability to produce cytokines CCL5 and CXCL1 (109). Preclinical studies show that the blocking of PGE2 in a murine model of metastatic breast cancer (122) and in human gastric cancer cells (123) restores NK cell function against tumor. Consistently, the efficacy of celecoxib, a cyclooxygenase-2 (COX2) inhibitor able to blockade PGE2 signaling, has been demonstrated in several clinical studies in many solid tumors (https://ClinicalTrials.gov).

The intracellular enzyme indoleamine 2,3-dioxygenase (IDO) is a critical regulator of the TME converting tryptophan into a number of metabolites with immunosuppressive function, including L-Kynurenine (124). IDO overexpression was associated with tumor progression and growth arrest of tumor-infiltrating NK cells (124, 125). L-Kynurenine is known to affect NK cell activity by interfering with the IL-2-driven upregulation of NKp46 and NKG2D (126). The simultaneous use of PGE2 and IDO inhibitors completely restored NK cell proliferation (116). The NK cell-mediated IFN-γ production in response to leukemic cells upregulates IDO, rendering tumor cells more resistant to NK cell-mediated killing (127).

Adenosine, a purine metabolite present at high concentration in the TME, also acts by limiting the activity of protective immune infiltrates, including NK cells, and enhancing that of Tregs and MDSCs (128). Adenosine accumulated in the TME by CD39 and CD73, causing inhibition of tumor-infiltrating NK cells by binding to the purinergic adenosine A_2A_ receptor expressed on cell surface (129–133). Reduction of adenosine by blocking both CD73 and the A_2A_ receptor was shown to affect tumor growth and promote recruitment of tumor-infiltrating NK cells (134).

Another factor causing dysfunction of tumor-infiltrating NK cells is hypoxia. Hypoxia downregulates NKp46, NKp30, NKp44, NKG2D, perforin, and granzyme B (135–138). Treatment with IL-2 restores NK cell cytotoxicity in multiple myeloma by increasing NKG2D expression (139).

Tumor cells secrete a large amount of lactate in the TME, causing acidosis and immunosuppression (140). Lactate accumulation has been associated with reduced NK cell cytotoxicity and downregulation of NKp46 (141, 142). The acidification of the TME has been shown to induce apoptosis of liver-resident NK cells in colorectal cancer liver metastases (143). The neutralization of the TME restored the cytotoxic activity of NK cells enhancing NKG2D expression (144).

Additionally, NK cells may express a wide range of immune checkpoint receptors that inhibit their function, including KIRs, CD94/NKG2A, PD1, CTLA4, TIM3, TIGIT, CD96, KLRG-1, LAG3, and, recently discovered, IL-1R8 (145). The effect of these immune checkpoint molecules on NK cell function has been reviewed (146, 147).

## Nk Cell-Based Therapeutic Strategies To Overcome Resistance In Solid Tumors

NK cell-based immunotherapy has been successfully applied for hematological malignancies and represents an attractive strategy even for treating solid tumors (148). The therapeutic approaches currently adopted are reported below.

### Use of Cytokines, Monoclonal Antibodies, and Immune Checkpoint Inhibitors

Therapeutic use of cytokines such as IL-12 and IL-18 in supporting ADCC (149–151), or IL-15 and IL-21 in triggering NK cell proliferation, NK-ARs induction, and cytokine production (152–156), resulted effective in increasing NK cell cytotoxicity against solid tumors. The cytokine agonists designed to improve the biological and pharmacokinetic activities of classical cytokines, including IL-15 superagonist ALT-803, showed promising results in both pre-clinical (157, 158) and clinical studies (159–161) (Table 1). Interestingly, encouraging results were obtained in combination with anti-cancer drugs, immune checkpoint inhibitors or vaccines, in terms of increased levels of circulating CD56^bright^ NK cells in ALT-803-treated patients.

**Table 1:** Some selected and most recent clinical trials (since January 1st 2016) on NK cell-based immunotherapies, in different forms of solid tumors, by using cytokines, monoclonal antibodies (mAbs), and immune checkpoint inhibitors.

**CYTOKINES SUPPORTING NK CELL ACTIVITY**				
**Cytokines**	In combination with^*^	**Phase**	**ClinicalTrial.gov identifier**	Tumor^**^
IL-15	–	I	NCT01946789	M, RCC, NSCLC, HNSCC
	–	I	NCT01572493	AST
	Ipilimumab and nivolumab	I	NCT03388632	MeM, RCC, NSCLC, HNSCC
IL-15 superagonist ALT-803	–	I	NCT02523469	NSCLC
	–	I	NCT03054909	OC
	Aldoxorubicin HCl	Ib/II	NCT03563157	CoC
		Ib/II	NCT03387098	PC
		Ib	NCT03586869	PC
		I/II	NCT03387085	TNIBC
	Bacillus Calmette-Guerin	II	NCT03022825	NMIBC
		I/II	NCT02138734	NMIBC
	Gemcitabine and nab-paclitaxel	I	NCT02559674	PC
	Anti cancer drugs	I/II	NCT03329248	PC
		I/II	NCT03175666	TNIBC
		I/II	NCT03136406	PC
	Nivolumab	I/II	NCT02523469	NSCLC
	Nivolumab or pembrolizumab or atezolizumab or avelumab	II	NCT03228667	NSCLC, SCLC, UC, HNSCC, MCC, M, RCC, GC, CeC, HC, MI, MRD, CoC
	ETBX-011 vaccine	Ib/II	NCT03127098	TC, CoC, OC, BC, LC, PC
**ANTIBODIES REDIRECTING NK CELL ACTIVITY**				
**Antibodies**	**In combination with**	**Phase**	**ClinicalTrial.gov identifier**	**Tumor**
Anti-ErbB3 (ISU104)	–	I	NCT03552406	AST, BC
Anti-globo H (OBI-888)	–	I/II	NCT03573544	LAMST
Anti-CD40 (JNJ-64457107)	–	I	NCT02829099	NSCLC, PC, M
Anti-CD47 (Hu5F9-G4)	–	I/II	NCT02953782	CoC
Anti-TAMUC1 (gatipotuzumab)	Anti-EGFR (tomuzotuximab)	Ib	NCT03360734	NSCLC, CoC, BC, GyC
Anti-semaforin 4D (pepinemab)	–	I/II	NCT03320330	OS
Anti-OX40 (INCAGNO1949)	–	I/II	NCT02923349	AST, EC, OC, RCC
Anti-GD2 (Ch14.18/CHO)	Nivolumab	I	NCT02914405	NB
	Nivolumab + ipilumumab + radiation	I/II	NCT03958383	M
**IMMUNE CHECKPOINT INHIBITORS**				
**mAbs targeting PD-1**				
Pembrolizumab	–	II	NCT03241927	M
	–	Ib	NCT03590054	MeM, HNSCC, UC, NSCLC, LC
	–	II	NCT02721732	AST
	–	II	NCT03428802	LAMST, OC, BC
	–	II	NCT03447678	NSCLC
	IL-12	I	NCT03030378	AST
	Autolous DC-CIK cells	I/II	NCT03190811	LC, RCC, GC, BlC, PC
	Lenalidomide	I	NCT02963610	Neoplams
	Enterococcus gallinarum (MRx0518)	I/II	NCT03637803	NSCLC, M, RCC, BlC
	Toll-like receptor (TLR) agonist (BDB001)	I/II	NCT03486301	AST
	AGEN1884	I/II	NCT02694822	AST
	Nab-paclitaxel + anti cancer drugs	II	NCT03289819	BC
	TLR9 agonist (AST-008)	I/II	NCT03684785	MeM, HNSCC, SCC, MCC
	Poly ICLC	I	NCT02834052	CoC
Toripalimab	–	I	NCT02857166	Neoplasms
	–	I	NCT03713905	Neoplasms
	–	I	NCT03474640	GC, NC, HC, EsC, STS, CoS
	–	I	NCT02836795	UrC
	Anti-VEGFR (surufatinib)	I	NCT03879057	NeT, LiC, GC
GLS-010	–	II	NCT03704246	Neoplasms
HX008	–	I	NCT03468751	Neoplasms
HLX10	–	I	NCT02715284	NSCLC, EC, MSI-H
TSR-042, dostarlimab	–	I	NCT03286296	LAMST, NSCLC
	–	I	NCT02715284	NSCLC, EC, MSI-H
GB226	–	I	NCT03374007	M, NSCLC, RCC, HNSCC, EC, LC, BC
INCMGA00012	–	I	NCT03059823	LAMST
AK105	–	I	NCT03352531	AST
SG001	–	I	NCT03852823	AST
CS1003	–	I	NCT03809767	Neoplasms
SCT-I10A	–	I	NCT03821363	Neoplasms
Sym021	Anti-LAG3 (Sym022) or anti-TIM3 (Sym023)	I	NCT03311412	AST
Tislelizumab	–	II	NCT03736889	MSI-H
	Anti-TIM-3 (BGB-A425)	I/II	NCT03744468	AST
	Anti-TIGIT (BGB-A1217)	I	NCT04047862	AST
	Anti PD-L1 (BGB-A333)	I/II	NCT03379259	AST
	PARP inhibitor (BGB-290)	I	NCT02660034	AST
Nivolumab	Metformin + rosiglitazone	II	NCT04114136	RCC, NSCLC, HCC, MSI-H, UC, GC, HNSCC
	Ipilimumab or BMS-986218	I/II	NCT03110107	AST
	Ipilimumab	II	NCT02834013	AST
	Anti-TIGIT (BMS-986207)	I/IIa	NCT02913313	AST
	Anti-TIGIT (OMP-313M32)	I	NCT03119428	Neoplasms
	Anti-LAG3 (relatlimab)	I	NCT02966548	AST
	Anti-VEGF (axitinib)	II	NCT03595124	RCC
	Relatlimab + ipilimumab	II	NCT04080804	HNSCC
	Relatlimab or ipilimumab + IDO1 inhibitor (BMS-986205)	I/II	NCT03459222	LAST
	Pembrolizumab + a protein kinase C inhibitor (trigriluzole)	II	NCT03229278	AST, RCC, HNSCC, NSCLC, BlC, M
	Ipilimumab or RGX-104 or docetaxel or pembrolizumab or carboplatin or demetrexed	I	NCT02922764	AST, NSCLC
	Adenoviral product (Ad-p53) + pembrolizumab or capecitabine	I/II	NCT02842125	AST, HNSCC
	Genetically modified HSV for tumor lysis (RP1)	I/II	NCT03767348	M, BlC, MRD, MSI-H
Avelumab	DC1c (BDCA-1) + myeloid DC + ipilimumab	I	NCT03707808	Neoplasms
	Anti-EGFR (cetuximab) + irinotecan	II	NCT03608046	CoC
ABBV-181	Anti-delta-like 3 protein (rovalpituzumab) or venetoclax	I	NCT03000257	NSCLC, TNIBC, OC, HC, GC, SCLC, Me, Cho, MCC, HNSCC
Sintilimab	IBI310	I	NCT03545971	AST
Spartalizumab, PDR001	Adenosine A2A receptor antagonist (NIR178)	II	NCT03207867	NSCLC, RCC, PC, UC, HNSCC, MSCC, TNIBC, M
SHR-1210	Parp inhibitor (SHR3162)	I	NCT03182673	AST
JNJ 63723283	Anti-FGFR1-4	I	NCT03547037	Neoplasms
AGEN2034	AGEN1884	II	NCT03894215	CeC
**mAbs targeting PD-L1**				
HLX20	–	I	NCT03588650	Neoplasms
KN035	–	I	NCT03248843	LAMST
	–	I	NCT03101488	HC
Atezolizumab	–	I	NCT02862275	Neoplasms
CS1001	–	Ia/Ib	NCT03312842	AST
MSB2311	–	I	NCT03463473	AST
SHR-1316	–	I	NCT03133247	AST
CK-301	–	I	NCT03212404	NSCLC, CC, HNSCC, M, RCC, UC, CoC, EC
LY3300054	CHK1 inhibitor (prexasertib)	I	NCT03495323	AST
Durvalumab	Tremelimumab	III	NCT03084471	Neoplasms
	Tremelimumab + fulvestrant	II	NCT03430466	BC
	Tremelimumab + azacitidine	Ib/II	NCT03019003	HNSCC
	Tremelimumab + radiation therapy	II	NCT03601455	BlC
	Tremelimumab + stereotactic body radiotherapy	Ib	NCT03275597	NSCLC
	Tremelimumab + metronomic vinorelbine	I/II	NCT03518606	BC, HNSCC, CeC, PrC
Avelumab	Anti-TNFRSF9 (utomilumab) + anti-OX40 (PF-04518600) + radiation therapy	I/II	NCT03217747	AST, PrC
**mAbs targeting CTLA-4**				
Fc-engineered IgG1 (AGEN1181)	–	I	NCT03860272	AST
Tremelimumab	PARP inhibitor (olaparib)	I/II	NCT02571725	OC
REGN4659	Cemiplimab	I	NCT03580694	NSCLC
INT230-6	Anti-PD-1	I/II	NCT03058289	M, HNSCC, BC, PC, LiC, CoC, LC, GB, BDC, OC, SCC
Ipilimumab	Oncolytic virus vaccine (pexa-Vec)	I	NCT02977156	Neoplasms
	SHR-1210	I	NCT03527251	NSCLC
	Nivolumab + anti cancer drugs	IIb	NCT03409198	BC
**mAbs targeting TIM-3**				
Sym023	–	I	NCT03489343	AST
INCAGNO02390	–	I	NCT03652077	CeC, GC, GC, EsC, HC, M, MCC, Me, NSCLC, Oc, HNSCC, RCC, MRD, UC
TSR-022	TSR-042 or anti-LAG3 (TRS-033)	I	NCT02817633	LAMST, CC, NSCLC
LY3321367	LY3300054	I	NCT03099109	AST
BGB-A425	Tislelizumab	I/II	NCT03744468	LAST
**BISPECIFIC mAbs**				
Anti-PD-1/anti-LAG3 (MDG013)	Anti-HER2 (margetuximab)	I	NCT03219268	Neoplasms
Anti-PD-1/anti-TIM3 (RO7121661)	–	I	NCT03708328	MeM, NSCLC, SCLC
Anti-PD-1/anti-CTLA-4 (AK104)	Oxaliplatin + capecitabine	I/II	NCT03852251	GC
Anti-PD-1/anti-CTLA-4 (MGD019 DART)	–	I	NCT03761017	AST
Anti-PD-L1/anti-TIM3 (LY3415244)	–	I	NCT03752177	AST
Anti-CTLA-4/anti-OX40 (ATOR-1015)	–	I	NCT03782467	AST
Anti-CTLA-4/anti-LAG3 (XmAb®22841)	Pembrolizumab	I	NCT03849469	AST
Anti-HER2 (MBS301)	–	I	NCT03842085	BC, SC
Anti-GD2/anti-CD3 (hu3F8-BsAb)	–	I/II	NCT03860207	NB, OS
**Fusion protein**				
Anti-PD-L1/TGFβRII (M7824)	–	I/II	NCT03436563	MSI-H RC, CoC

* Immune checkpoint inhibitors reported in the column indicating the “combination” treatment: ipilimumab, pembrolizumab, AGEN1884, IBI310, tremelimumab, BMS-986218: anti-CTLA-4; nivolumab, pembrolizumab, TRS-042, tislelizumab, cemiplimab: anti-PD-1; atezolizumab, avelumab, durvalumab, LY3300054: anti-PD-L1.

** *Abbreviation of solid tumors: AR, adenocarcinoma of rectum; AST, advanced solid tumors; BC, Breast Cancer; BlC, bladder cancer; BDC, bile duct cancer; BTC, biliary tract cancer; CeC, Cervical Cancer; Cho, cholangiocarcinoma; CoC, Colorectal Cancer; CoS, chondrosarcoma; EC, endometrial cancer; EsC, esophageal cancer; ES, Ewing's sarcoma; ESCC, esophageal squamous cell carcinoma; FTC, fallopian tube cancer; GC, Gastric Cancer; GyC, gynecological cancers; GB, glioblastoma, HC, Hepatocellular Carcinoma; HNSCC, Head and Neck Squamous Cell Carcinoma; HRM, high risk melanoma; LAMST, locally advanced or metastatic solid tumors; LaC, laryngeal cancer; LC, Lung Cancer; LiC, liver cancer; M, Melanoma; MCC, Merkel Cell Carcinoma; MGB, malignant glioma of brain; Me, mesothelioma; MEC, metastatic esophageal cancer; MeM, metastatic melanoma; MI, Microsatellite Instability; MRD, Mismatch Repair Deficiency; MSC, metastatic solid cancer; MSCC, microsatellite stable colon cancer; MSI-H, non-endometrial deficient mismatch repair (dMMR)/microsatellite instability-high; NC, nasopharyngeal carcinoma; NeT, neuroendocrine tumors; NB, neuroblastoma; NMIBC, non-muscle invasive bladder cancer; NSCLC, Non-Small Cell Lung Cancer; OC, Ovarian Cancer; OS, osteosarcoma; PC, Pancreatic Cancer; PDCC, pancreatic ductal cell carcinoma; PhC, pharyngeal cancer; PPC, primary peritoneal cancer; PrC, prostate carcinoma; RCC, Renal Cell Carcinoma; RS, rhabdomyosarcoma; SC, stomach cancer; SCC, squamous cell carcinoma; SCLC, Small Cell Lung Cancer; STS, soft tissue sarcoma; TC, Thyroid Cancer; TNIBC, triple-negative invasive breast carcinoma; ToC, tongue cancer; UC, Urothelial Carcinoma; UrC, urological cancer. Abbreviation of cells: DCs, dendritic cells; CIK, Cytokine-induced killer cells*.

The secretion of ligands for NK-ARs by solid tumor cells has been associated to immune suppressive effects (162). In a murine model, the neutralization of soluble NKG2D ligands such as MICA and MICB with mAb B10G5 was effective against prostate carcinoma and metastasis, leading to the enhanced NK cell infiltration in the tumor parenchyma (163), and improving CTLA-4 blockade therapy (164).

Another approach to sensitize solid tumors to NK cell-mediated lysis is to improve ADCC with monoclonal antibodies (mAbs) specific for tumor antigens. Several clinical trials exploring the efficacy of various mAbs targeting tumor antigens are in phase I and II (Table 1). This therapeutic approach is very promising even in challenging pediatric tumors, such as neuroblastoma, in which amplification of the *MYCN* oncogene, clinically associated with poor prognosis, has been correlated with the reduced tumor susceptibility to NK cell-mediated killing (165).

The adoption of the chimeric mAb Ch14.18 recognizing the main tumor antigen GD2 on neuroblastoma cells was very successful (166) and presently under investigation in several clinical trials in combination with immune checkpoint inhibitors or in the form of chimeric antigen receptors (CARs) for both CAR-T (167) and CAR-NK cells (Table 1).

On the other hand, several approaches aimed to dampen the NK cell-inhibitory signals, such as those mediated by immune checkpoint molecules, have been explored, thus triggering directly immune effector functions including those mediated by NK cells. The initial adoption of anti-KIRs reported very few positive results (168), with potential benefits when used in combination with anti-PD1 for the cure of non-small cell lung cancer patients (169). Blocking of PD1/PD-L1 signaling enhances the production of cytokines and degranulation of NK cells *in vitro* by reducing their apoptosis (170). In a xenograft model, anti-PD1 suppressed the tumor growth of digestive cancers in an NK cell-dependent manner, suggesting a crucial role of PD1 in NK cell function (170). Moreover, anti-CTLA-4 combined with IL-15/IL-15Rα enhances the NK cell tumor infiltration, improving the tumor growth control in xenograft murine models of solid tumors. Melanoma patients treated with anti-CTLA-4 (ipilimumab) had higher intratumoral CD56 expression (171). Consistently, several phase I and II clinical trials are ongoing to test the efficacy of different anti-PD1, anti PD-L1, and anti-CTLA-4 mAbs, alone or in combination with other Abs, in multiple forms of advanced and metastatic solid tumors (Table 1). The competition of TIGIT and CD96 binding to DNAM-1 ligands PVR and Nectin-2 renders tumor-infiltrating NK cells exhausted (172–174). TIGIT-targeting therapy represents a promising cure for solid tumor (172, 173, 175). In preclinical studies, the murine model anti-TIGIT is able to improve the antitumor effect of anti-HER2 mAb (176) alone, and in combination with PD1/PD-L1 inhibitors (176). Preclinical studies showed that CD96 blocking combined with anti-PD1 or anti-CTLA-4 enhances NK cell infiltration and IFN-γ production, thus reducing tumor lung metastases (177). The blockade of TIM-3 was also found to enhance NK cell function against melanoma cells (178). The therapeutic efficacy of the combined use of different immune checkpoint inhibitors, such as anti-TIM-3 and anti-TIGIT, is currently tested in ongoing phase I and II clinical trials in solid tumor patients (Table 1).

Different bispecific mAbs for immune checkpoint inhibitors and for GD2–CD3 are in phase I and II clinical trials, respectively (Table 1). In addition, various bispecific NK cell engaging antibodies, such as ErbB2-CD16, EpCAM-CD16, and HER2-CD16, were promising for solid tumors in preclinical studies (179–181). Interestingly, the combined use of antibodies with cytokines, such as anti-GD2/GM-CSF/IL-2 (182, 183), as well as the adoption of the novel fusion proteins by linking antibodies to cytokines, such as anti-GD2 to IL-15 superagonist (184), or the TNF-targeting human IgG1 NHS76 to IL-12 (185), reported great therapeutic results. Currently, the fusion protein anti-PD-L1/TGFβRII is in phase I/II clinical trial (Table 1). The use of a novel monomeric carcinoembryonic-antigen (CEA)-targeted immunocytokine, or cergutuzumab amunaleukin (CEA-IL2v, RG7813), has shown success in preclinical models of colon cancer, resulting in a large expansion of NK cells and activation of T cells (186).

### Adoptive Transfer of NK and CAR-NK Cells

Recently, great interest in the treatment of solid tumors is focused on the adoptive transfer of *ex vivo* expanded and activated NK cells that, for their peculiar innate features, relatively short lifespan, low risk of overexpression in infused patients, higher safety compared to infused T cells, and low costs, could represent an optimal therapeutic strategy (187, 188). Activated and expanded NK cells can be obtained by different sources, including NK cell lines, primary NK cells, umbilical cord blood (UCB)- and induced pluripotent stem cell (iPSC)-derived NK cells (189) (Table 1). Of note, NK cells are tolerant to health cells and sensible to tumor cells, mostly in alloreactive conditions (190). Thus, haploidentical allogenic NK cells represent an optimal cellular immunotherapy product, mainly for immunocompromising diseases, including solid tumors, whose patients can scantly count on their own cells and often need donor NK cells (191–193). Interestingly, there are several clinical trials in phase I and II in progress to treat solid tumors based on NK cell adoptive transfer combined with ALT-803, mAbs, anti-cancer drugs, irreversible electroporation, and cryosurgery (Table 2). This approach could overcome the effects of tumor immune evasion (194, 195) since mature and activated NK cells provided by current protocol of iPSC-derived NK cell expansion are able to infiltrate and kill solid tumors. This was firstly evaluated in a murine xenograft model of ovarian cancer (196), and more recently in subjects with advanced solid tumors as monotherapy and in combination with mAb (NCT03319459) or antitumor drugs (NCT03213964) (Table 2). Of interest, among most efficient immune-modulatory drugs able to induce ligands for NK-ARs on cancer cells (197), lenalidomide is used in a clinical setting in combination with expanded and activated NK cells in some hematopoietic malignancies and neuroblastoma patients (NCT02573896).

**Table 2:** Some selected and most recent clinical trials (since January 1st 2016) on NK cell-based immunotherapies, in different forms of solid tumors, by using adoptive transfer of NK and CAR-NK cells.

**ADOPTIVE TRANSFER OF *EX VIVO* EXPANDED AND ACTIVATED NK CELLS**				
**NK cell source**	**In combination with**	**Phase**	**ClinicalTrial.gov identifier**	**Tumor**
NK-92	ALT-803	II	NCT02465957	MCC
Autologous vs. allogenic	–	II	NCT02853903	Neoplasms
Autologous	–	E I	NCT03662477	LC
	–	II	NCT03410368	NSCLC
	Bortezomib	I	NCT00720785	PC, CoC, NSCLC
	Anti cancer drugs	II	NCT02734524	NSCLC
	Anti-GD2 (Ch14.18) + lenalidomide	I	NCT02573896	NB
	Sintilimab	II	NCT03958097	NSCLC
Allogenic	–	I	NCT03358849	BTC
	ALT-803	I	NCT02890758	CoC, AR, STS, ES, RS
	Anti-GD2 (Hu14.18)-IL2 fusion protein	I	NCT03209869	NB
	Anti-GD2 + IL2	I/II	NCT03242603	NB
	Anti-GD2 (Hu3F8) + rIL2	I	NCT02650648	NB
	Pemetrexed	I	NCT03366064	NSCLC
NK cells *(not specified)*	–	I	NCT03619954	AST, OC
	Trastuzumab	I/II	NCT02843126	BC
	Cetuximab	I/II	NCT02845856	NSCLC
	Anti-VEGF (bevacizumab)	I/II	NCT02857920	Neoplasms
	Nivolumab	I/II	NCT02843204	Neoplasms
	Irreversible electroporation (IRE)	I/II	NCT02718859	PC
		I/II	NCT03008343	LiC
	Cryosurgery	I/II	NCT02843802	LiC
		I/II	NCT02844335	BC
		I/II	NCT02849379	ToC
		I/II	NCT02849314	LaC
		I/II	NCT02849327	PhC
		I/II	NCT02849340	CeC
		I/II	NCT02849015	LiC
		I/II	NCT02843581	MEC
		I/II	NCT02849353	OC
		I/II	NCT02843815	NSCLC
		I/II	NCT02843607	RCC
		I/II	NCT02849366	RS
	NKT cells	I	NCT03198923	NSCLC
CIK	DCs	I/II	NCT03047525	CcC, RC, NC, LC
UCB-derived	–	I/II	NCT03634501	LC, BC, CoC, PC, OC
	Anti cancer drugs	I	NCT03420963	AST
		I	NCT03539406	OC
iPSC-derived (FATE-NK100)	IL-2	I	NCT03213964	OC
	Anti-HER2/neu and anti-EGFR	I	NCT03319459	BC, CoC, HNSCC, HC, NSCLC, RCC, PC, M
iPSC-derived (FT500)	–	n. i.	NCT04106167	Neoplasms
	Immune checkpoint inhibitors	I	NCT03841110	Neoplasms
**ADOPTIVE TRANSFER OF CAR-NK CELLS**				
**CAR NK cells**	**In combination with**	**Phase state**	**ClinicalTrial.gov identifier**	**Tumor**
CD16A-IL2-NK-92 (haNK)	–	I	NCT03027128	LAST
	IL-15 superagonist (N-803) and avelumab	II	NCT03853317	MCC
	Anti-cancer drugs, vaccines and immune checkpoint inhibitors	I/II	NCT03387111	SCC, neoplasms
ROBO1-NK cells	–	I/II	NCT03940820	Neoplasms
BiCAR-NK cells (ROBO1 CAR-NK cells)	–	I/II	NCT03941457	PC
ErbB2/HER2-NK (NK-92/5.28z)	*Intracranial application*	I	NCT03383978	GB
NKG2D-NK	IL-2	I	NCT03415100	Neoplasms, OC
MUC1-NK	–	I/II	NCT02839954	HC, NSCLC, PC, TNIBC, MGB, CoC, GC

Abbreviation of solid tumors and immune checkpoint inhibitors reported in the column indicating the “combination” treatment: see Table 1 legend.

*Abbreviation of cells: DCs, dendritic cells; CIK, Cytokine-induced killer cells; NKT, natural killer-T cells; iPSC, induced pluripotent stem cells; UCB, umbilical cord blood. Other abbreviations: E I, early I phase; n. i., non-interventional, observational study*.

In order to potentiate both the specificity and activity of NK cells against solid tumors, genetically modified NK cells have been produced and clinically adopted with promising results. NK cells obtained by different sources have been engineered for CARs able to recognize specific tumor antigens or ligands for activating receptors (198–201). To avoid the need of exogenous IL-2 in culture, high-affinity NK (haNK) cells were obtained by engineering the NK-92 cell line for the expression of CD16A and IL-2 (202) and used in patients with solid tumors (NCT03027128). Several CAR-engineered NK cells recognizing EGFR for breast cancer brain metastases (203), both ErbB2/HER2 (204, 205) (NCT03383978) upon intracranial injection, and EGFRvIII (206) for glioblastoma, GD2 for neuroblastoma (207), EpCAM for breast carcinoma (208), NKG2D for ovarian cancer (201), and both MUC1 and ROBO1 for advanced refractory solid tumors, have been produced for preclinical studies and phase I and II clinical trials (Table 2). Finally, of emerging interest are the extracellular vesicles (EVs) secreted by large-scale *in vitro* expanded NK cells, that, containing lytic protein, showed antitumor efficacy not only in malignant hematopoietic cell lines but also in neuroblastoma and breast carcinoma cell lines, and in a xenograft murine model of glioblastoma (209, 210).

## Perspectives

NK cells play a crucial role in triggering antitumor immune response. Although high levels of tumor infiltrating NK cells are associated with a better prognosis in certain human solid tumors, the immunosuppressive TME weakens their function in favor of neoplastic progression. Understanding the mechanisms adopted by the TME to hinder the NK cell function and how they can be neutralized is of fundamental importance to develop effective anti-cancer therapeutic protocols. In this regard, the use of NK cell-based immunotherapy, by the combined use of cytokines, mAbs, immune checkpoint inhibitors, and the adoptive transfer of NK cells and CAR-NK cells, appears to be promising in the treatment of solid tumors.

A significant number of cancer immunotherapies that involved engineering NK cells before adoptive transfer into patients have been developed. There is the possibility of engineering NK cells to make them resistant to the metabolically restrictive TME as well as to immunosuppressive molecules generated by the tumor and TME.

Overall, it is now clear that TME is crucial for the normal function of NK cells and that future investigations and pre-clinical studies in this area are likely needed to fully discern the biology of NK cells and reveal new and exciting anti-cancer therapeutic opportunities.

## Author Contributions

All the authors edited the manuscript and approved the final version for publication.

### Conflict of Interest

The authors declare that the research was conducted in the absence of any commercial or financial relationships that could be construed as a potential conflict of interest.
